# Synthesis, antiasthmatic, and insecticidal/antifungal activities of allosamidins

**DOI:** 10.1080/14756366.2019.1623208

**Published:** 2019-07-16

**Authors:** Gangliang Huang, Hualiang Huang

**Affiliations:** a Chongqing Key Laboratory of Green Synthesis and Application, Active Carbohydrate Research Institute, Chongqing Normal University, Chongqing, China;; b School of Chemistry and Environmental Engineering, Wuhan Institute of Technology, Wuhan, China

**Keywords:** Allosamidins, synthesis, antiasthmatic activity, insecticidal/antifungal activities

## Abstract

Allosamidins come from the secondary metabolites of *Streptomyces* species, and they have the pseudotrisaccharide structures. Allosamidins are chitinase inhibitors that can be used to study the physiological effects of chitinases in a variety of organisms. They have the novel antiasthmatic activity and insecticidal/antifungal activities. Herein, the synthesis and activities of allosamidins were summarized and analyzed.

## Introduction

**Figure 1.**
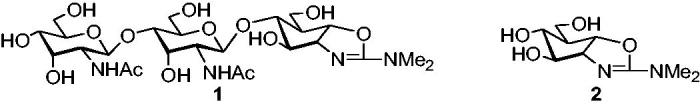
Structures of allosamidin **1** and allosamizoline **2**.

An effective chitinase inhibitor was found in screening metabolites of actinomycetes, which was isolated from the mycelium extract of *Streptomyces* 1713^1^^,^^2^. Its new structure was elucidated, which was a pseudotrisaccharide containing two β-linked *N*-acetyl-2-amino-2-deoxy-D-allopyranoside building blocks. The new disaccharide is linked to allosamizoline **2** (Figure 1) through its reducing terminal. It is a new family-18 chitinase inhibitor, named allosemidin **1**. The compound has a unique chemical structure and its synthetic method is very challenging^3–29^. The selection of glycosylation methods to assemble the structural units of allosamidin and its analogues is the essential difference between each reported total synthesis method. The main goal of these glycosylation methods is to produce β-configuration products. This article analyses the synthesis and activity of isoamides. The synthesis and activities of allosamidins were reviewed herein.

## Preparation of allosamizoline 2

Allosamizoline **2** is an important unit of allosamidin **1**. Compound **2** and its analogues were mainly synthesized with non-sugar compounds as raw materials^30–43^. Sugar was also used in the synthesis of compound **2**^44–47^. Under proper protection, sugar-based receptors were prepared first, and then combined with the required oligosaccharide donors to complete the synthesis of compound **1**.

**Scheme 1.**
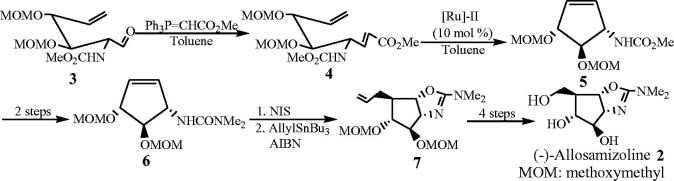
Preparation of (-)-allosamizoline **2**.

The regio- and stereocontrolled total synthesis of (-)-allosamizoline **2** was studied (Scheme 1)^48^. Using D-glucosamine as raw material, aldehyde **3** was synthesized by five-step reaction. The Wittig olefination of aldehyde **3** was carried out by using ylide Ph_3_P = CHCO_2_Me to obtain a high yield (91%) of acrylate **4**. In the ring-closed metathesis reaction, the terminal substituent of the alkene was not transferred to the cyclized product. The ring-closing metathesis also took place smoothly and cyclopentene **5** was obtained in 88% yield. The key steps of the synthesis included halogen cyclization to provide oxazoline ring, followed by stereoselective addition of alkene radical, and finally carried out alkene isomerization to form hydroxymethyl. (-)-Allosamizoline **2** was prepared by 13-step reaction with an overall yield of 22%.

**Scheme 2.**
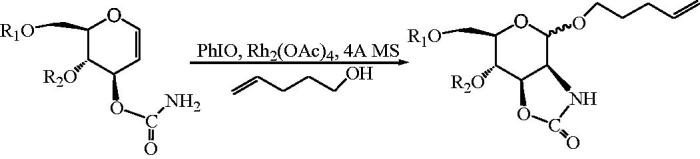
Preparation of mannosamine derivatives with oxazolidinone protection.

Rhodium-catalyzed oxidative cyclization of glucal 3-carbamates to oxazolidinone-protected mannosamine derivatives (Scheme 2)^49^ could be used to synthesize various allosamidin analogues. The stereoselectivity of anomeric centers relied on the properties of protective groups and solvents. It was proved that benzyl protection mainly produced a product of α-configuration. Solvents with lower polarity, such as hexane and benzene, also increased the anomeric proportion. Its formation was with a primary amide containing carbamate as a raw material.

## Synthesis of allosamidin compounds

Solid phase synthesis is a fast and effective method for the synthesis of oligosaccharides^50^^,^^51^. In the multi-step solid phase synthesis of oligosaccharides, excess reactants or by-products can be easily removed. Oligosaccharide synthesis is important for glycosylation, which involves sugar-based donors and receptors. For example, Huang's group first synthesized the α-trichloroacetimidate donors **8**, **9** and allosamizoline-derived acceptor **10**. Moreover, the solid-state synthesis of allosamidin **1** was developed^52^.

**Scheme 3.**
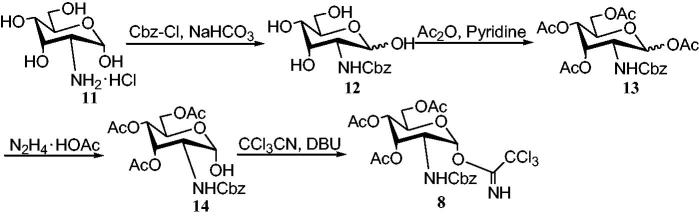
Synthesis of donor **8** with *N*-Cbz protection.

To synthesize α-trichloroacetimidate donor **8** (Scheme 3), the preparation of α-D-allosamine-hydrochloride **11** was carried out^53^. Compound **11** was treated with benzyloxycarbonyl (Cbz)-Cl and NaHCO_3_/H_2_O, *N*-benzyloxycarbonyl protected allosamine **12** in 85% yield was obtained. Compound **12** was acetylated in pyridine to obtain the α/β isomer (4:1) mixture of tetraacetate **13**. The anomeric acetyl group was selectively removed in N,N-Dimethylformamide (DMF) with hydrazine acetate to obtain the hemiacetal **14**. In the presence of 1,8-diaza[5.4.0]bicycloundec-7-ene (DBU), the compound 14 was reacted with the CCl_3_CN to obtain 82% yield of α-trichloroacetimidate donor **8**.

**Scheme 4.**
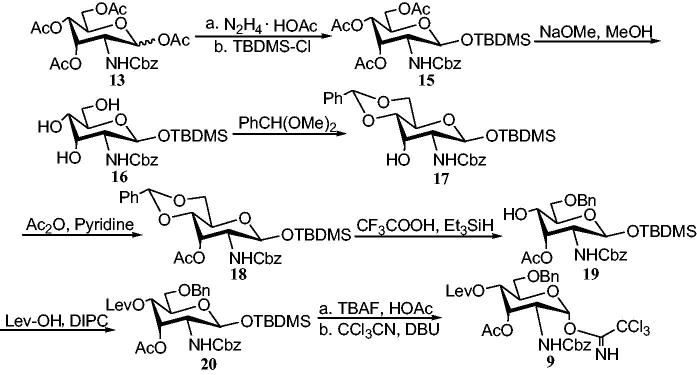
Synthesis of donor **9** with *N*-Cbz protection.

The hemiacetal **14**, which was directly carried out without purification, was produced in DMF with the hydrazine acetate-treated compound **13**. Compound **14** was reacted with *tert*-butyldimethylsilyl (TBDMS)-Cl in imidazole to give a β-configuration TBDMS derivative **15**. A 95% yield of TBDMS 2-deoxy-*N*-benzyloxycarbonylamino-β-D-allopyranoside **16** was obtained by deacetylation of compound **15** with NaOMe/MeOH. Compound **16** was treated with benzaldehyde dimethylacetal to obtain 4,6-*O*-benzylidene derivative **17**. The reaction of compound **17** and Ac_2_O was carried out in the presence of pyridine to give acetate **18** in a yield of 94%. 6-*O*-Bn acceptor **19** was obtained by regioselective reduction of benzylidene acetal **18** with CF_3_COOH/Et_3_SiH at 0 °C in a yield of 86%. In the presence of *N,N*′-diisopropylcarbodiimide (DIPC), compound **19** was reacted with levulinic acid to give the orthogonally protected allosamine **20** in 95% yield. In the presence of acetic acid, tetrabutylammonium fluoride (TBAF) was used to remove the anomeric TBDMS group. The crude product was then reacted with the CCl_3_CN and DBU to give the α-trichloroacetimidate donor **9** (Scheme 4).

**Scheme 5.**
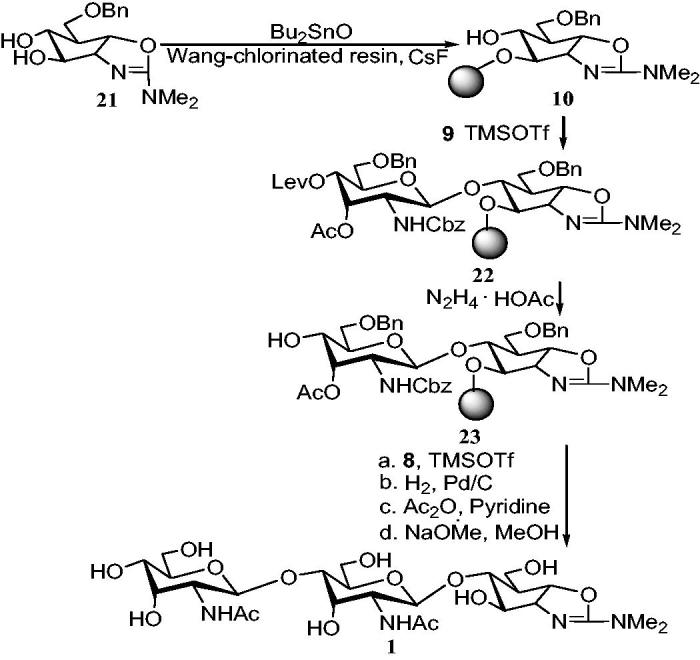
Solid phase synthesis of allosamidin **1**.

Diol **21** was prepared (Scheme 5)^54^. Compound **21** was selectively benzylated on C-3 hydroxyl group by the method of stannylene^55^ to produce the dibenzylated unit **10** in a yield of 45%. The glycosylation was carried out by using 3.0 equivalent donor and 1.2 equivalent trimethylsilyl trifluromethanesulfonate (TMSOTf) as promoter to activate trichloroacetimidate donor. TMSOTf promoted the glycosylation of trichloroacetimidate donor **9** with 6-*O*-benzylallosamizoline alcohol acceptor **10** at low temperature. The corresponding β-pseudodisaccharide **22** was obtained with a yield of 68%. Wang resin was removed from building block **22** with trifluoroacetic acid. The product was analyzed by high pressure liquid chromatography (HPLC). The receptor **23** was obtained by cleaving the levulinoyl ester with hydrazine acetate dissolved in methanol. After the glycosylation was carried out with acceptor **23** and donor **8**, the resin was washed, filtered, and dried in vacuum for 12 h. Sugar-based resin were catalytically hydrogenated to decompose Cbz, Wang resin, and Bn in a yield of 90%. The mixture was acetylated with Ac_2_O-pyridine and then deacetylated with NaOMe-MeOH to provide the crude product. The crude product was purified by size-exclusion chromatography on Biogel P4 to obtain the corresponding target pseudotrisaccharide **1** and the final three-step yield was 71%.

**Scheme 6.**
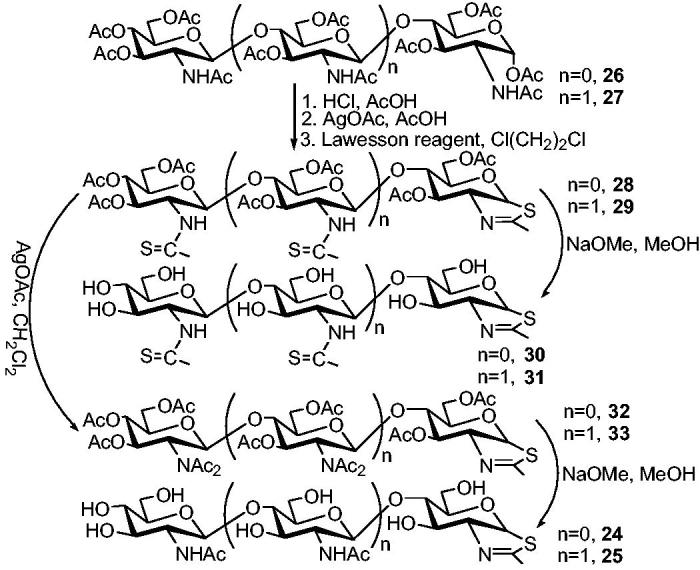
Synthesis of chitobiose and chitotriose thiazolines (**24** and **25**), and their thioamide analogues (**30** and **31**).

An international group has developed a new type of chitinase inhibitor^56^. The core building block is a cyclic sugar fused with thiazoline, a five-membered ring consisting of one N, one S and three C atoms (Scheme 6). This arrangement simulates a ring intermediate product formed during chitinase degradation and interacts with the binding sites on chitinase. To enhance the inhibition, the researchers added two or three additional sugar building blocks, similar to chitin (chitobiose or chitotriose).

The synthesis of disaccharide and trisaccharide thiazolines **24** and **25** began with octaacetylchitobiose **26** and undecaacetylchitotriose **27** in turn (Scheme 6)^56^. The α-configuration of acetoxy groups of compounds **26** and **27** was reversed to give the corresponding β-anomers, the anomeric chlorides were obtained by the initial treatment with HCl and AcOH, and then treated with AgOAc/AcOH. After treatment with Lawesson reagent, thiazolines **28** and **29** were obtained by affecting both the conversion of amides to thioamides and the intramolecular substitution of adjacent thioamide sulfur atom to the anomeric β-acetoxy group. The partial deacetylation of per-O-acetylated thiazolines **28** and **29** gave two additional chitinase inhibitors, namely the chitobiose thiazoline thioamide **30** in a yield of 89% and chitotriose thiazoline dithioamide **31** in a yield of 80%. To synthesize target compounds **24** and **25**, thioamides **28** and **29** were converted to diacetylimides **32** and **33** (81% and 60% yields in turn) with silver acetate/dichloromethane without destroying the thiazoline part. Finally, chitobiose thiazoline **24** (69% yield) and chitotriose thiazoline **25** (78% yield) were obtained by O-deacylation and mono-N-deacylation of imides **32** and **33** with sodium methanol/methanol.

**Scheme 7.**
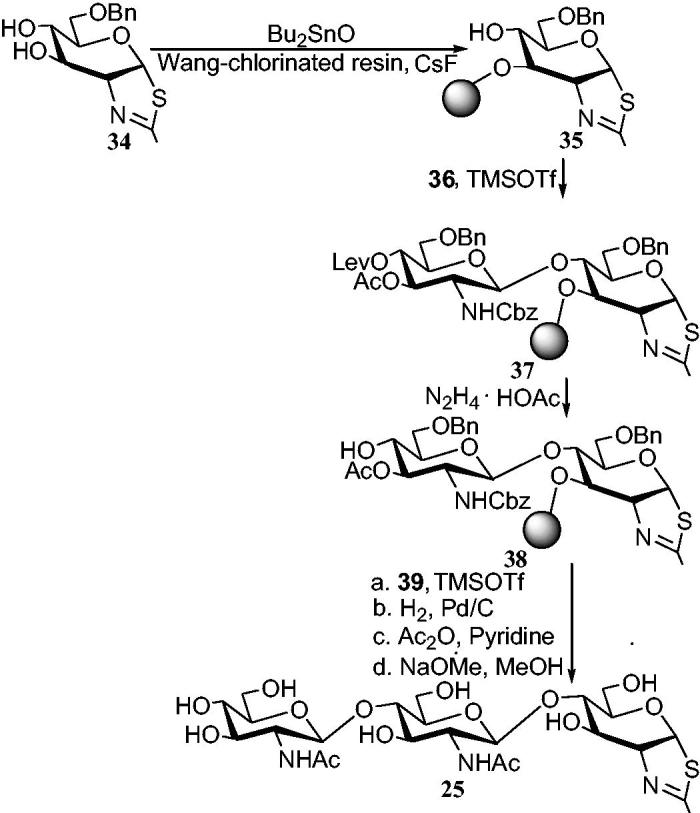
Solid phase synthesis of chitotriose thiazoline **25**.

It was indicated that chitobiose and chitotriose thiazolines (**24** and **25**) were synthesized by traditional method. As above-mentioned, the of allosamidin **1** was synthesized by solid phase method. So, compound **25** was successfully synthesized by the similar approach (Scheme 7)^57^. Compounds **36** and **39** were used as the corresponding α-trichloroacetimidate donors.

GlcNAc thiazoline is a poor chitinase A (ChiA) inhibitor whose *K*_i_>1 mM. However, adding one GlcNAc residue to compound **24** increased the binding power by at least 40 times, and the second GlcNAc residue further increased the affinity by 100 times. The *K*_i_ value of pseudotrisaccharide **25** was much lower than that of allosamidin (*K*_i_=0.6 μM) in inhibition of ChiA^56^. This result contrasted with recent finding that the disaccharide thiazoline with sulfur linkage was not a significant ChiA inhibitor. No significant inhibition was observed in this study might be due to the different geometric structure imposed by the thioglycosidic linkage^58^.

The main methods of synthesis of compound **25** were compared as shown in Table 1.

**Table 1.** Comparison of the three synthetic methods of compound **25**.

Comparison		
Synthetic method of compound **25**	Number of synthetic steps	Number of column chromatography separation
Solid–liquid phase	7	3
Total solid phase	8	0
Total liquid phase	6	6

## The activities, binding mode to chitinases and structure-activity relationships of allosamidins

### The activities of allosamidins

In mouse asthma model, allosamidin showed antiasthmatic activity^59^ and decreased inflammatory symptoms were observed in rabbits with endotoxin-induced uveitis^60^. Demethylallosamidin, a derivative of allosamidin, had strong inhibitory activity on yeast chitinase^61^ and human chitosidase^62^. The inhibitory effect of demethylallosamidin on acidic mammalian chitinase (AMCase) was stronger than that of allosamidin and had strong anti-asthma activity. Demethylallosamidin inhibited IL-13-induced hyperresponsiveness and had better potential as an anti-asthma drug than allosamidin^63^. There is a need to use other target molecules in the future to investigate the difference in the anti-asthma activity of allosamidin and demethylallosamidin.

Allosamidins can inhibit chitinase activity. So, they can prevent the ecdysis of insect larvae and pupae, and isolation of fungal microspore mother cells. As a result, they play a role in insecticidal and antifungal activities^64^. At very low concentrations, pseudo-trisaccharide allosamidin **1** has competitive inhibitory activity against chitinases. The injection of compound **1** into silkworm (*Bombyx mori*) larvae and armyworm (*Leucanobia separata*) strongly interfered with larval molting and increased the mortality of lepidopterous pests. The inhibitory effects of allosamidin **1** on *Bomhyx mori* larvae and *Leucania separata* larvae were EI_50_ = 2 μg and 4 μg, respectively^1^. EI_50_ is a 50% molting inhibition. Compound 1 and its derivatives could significantly increase the mortality of fly larvae (*Lucilia cuprina*) after exposure or feeding test. Allosamidin **1** could result in larval mortality in the webbing clothes moth *Tineola bisselliella* because of severe morphological alterations, namely delaying growth and interrupting molting. This occured during larval development. Compound **1** could also induce the killing effect of aphids, increase larval mortality and decrease the reproductive ability of aphid *Myzus persicae*^65^.

Allosamidin **1** has the broad-spectrum chitinase inhibitory activity. Compound 1 and its derivatives, i. e., methylallosamidin, demethylallosamidin, glucoallosamidin A, glucoallosamidin B, and methyl-*N*-demethylallosamidin^66^, could also kill different human pests and pathogens, such as plasmodium^67,68^ and nematode^69^. Allosamidin **1** also had antibacterial^70^ and insecticidal/antifungal activities^71^. In *Streptomyces* species producing compound **1**, this inhibitor was beneficial to the production of chitinase insensitive to compound **1**, which was beneficial to fungal growth^72^.

### The binding mode to chitinases of allosamidins

According to the NMR spectrum information, ab initio calculations and the spatial squeezing effect between molecules, it could be proved that the binding power in the allosamizoline part of allosamidin **1** was the strongest^73^.

The pseudotrisaccharide allosamidin is an effective family-18 chitinase inhibitor, which has obvious biological activity against insects, fungi and Plasmodium falciparum, and affects their life cycle. Similar to other chitinases, demethylallosamidin derivatives have a 10-fold inhibitory effect on human chitinase. These structures explained the effects of changing hydrogen bonds and hydrophobic interactions as well as the effect of substituted water molecules on the inhibition^62^.

Allosamidin **1** is located in the deep active site of ChiA from *S. marcescens* and interacts with three important residues: Glu315 is the catalyzed proton donor. Asp313 takes two conformations in the primary structure, but faces toward Glu315 in the inhibitor complex. Tyr390 is located opposite Glu315 in the active site tunnel^74^.

The inhibition of the family-18 chitinase is becoming a target for pest and fungal control and the treatment of asthma and inflammation. Under the condition of pH6.0, the binding of allosamidin **1** required the deprotonation of Asp142-Glu144 catalytic diad^75^.

### The structure-activity relationships of allosamidins

The structure-activity relationship of allosamidins is as follows. Allosamidins can inhibit the enzymes of GH18 and GH20 families. NAG-thiazoline is a potential inhibitor of *N*-acetylhexosaminase of GH20 family. It is introduced into the structure of allosamidin analogues. The obtained compounds have been proved to have good inhibitory activity against 18 family chitinases. Substituted *N*-glycosyl oxazolines, *N*-glycosyl aminoxazolines, and *N*-glycosyl thiazolines also exhibit enzymatic inhibition. Allosamidins containing *N*-acetylglucosamine structural unit have the good inhibitory effect on chitinases. In addition, the side chain groups of allosamizoline analogous structural units can be extended appropriately, but if the side chain groups are prolonged too much, the volume of side chain groups will be too large and the steric hindrance will increase, which will reduce the affinity to 20 family glycosylhydrolases^76^.

## Conclusion

To sum up, the synthesis efficiency of allosamizoline **2** and its analogues can be greatly improved by introducing related metal catalytic reactions, which is beneficial to improve the selectivity of the reactions. At the same time, it is proved that the synthesis efficiency of allosamidin **1** and its analogues can also be improved by solution phase synthesis and solid phase synthesis. The activities of allosamidin **1** and its analogues showed that they can not only be used as lead compounds to develop the effective anti-asthma drugs and insecticidal/antifungal agents, but also as probes to investigate the physiological effects of chitin-like proteins. The development trend in this field is to develop new synthesis methods, improve synthesis efficiency, and screen out new allosamidins which can significantly inhibit chitinases and have high anti-asthma and insecticidal/antifungal activities.
